# California's COVID-19 Virtual Training Academy: Rapid Scale-Up of a Statewide Contact Tracing and Case Investigation Workforce Training Program

**DOI:** 10.3389/fpubh.2021.706697

**Published:** 2021-08-09

**Authors:** Debbie B. Brickley, Maeve Forster, Amelia Alonis, Elizabeth Antonyan, Lisa Chen, Alicia DiGiammarino, Alina Dorian, Caitlin Dunn, Alice Gandelman, Mike Grasso, Alice Kiureghian, Andrew D. Maher, Hannah Malan, Patricia Mejia, Anna Peare, Michael Prelip, Shira Shafir, Karen White, Rachel Willard-Grace, Michael Reid

**Affiliations:** ^1^Institute for Global Health Sciences, University of California, San Francisco, San Francisco, CA, United States; ^2^Curry International Tuberculosis Center, University of California, San Francisco, San Francisco, CA, United States; ^3^Department of Community Health Sciences, Fielding School of Public Health, University of California, Los Angeles, Los Angeles, CA, United States; ^4^Center for Excellence in Primary Care, University of California, San Francisco, San Francisco, CA, United States; ^5^California Prevention Training Center, University of California, San Francisco, San Francisco, CA, United States

**Keywords:** COVID-19, case investigation, contact tracing, workforce, public health preparedness

## Abstract

Case investigation (CI) and contact tracing (CT) are key to containing the COVID-19 pandemic. Widespread community transmission necessitates a large, diverse workforce with specialized knowledge and skills. The University of California, San Francisco and Los Angeles partnered with the California Department of Public Health to rapidly mobilize and train a CI/CT workforce. In April through August 2020, a team of public health practitioners and health educators constructed a training program to enable learners from diverse backgrounds to quickly acquire the competencies necessary to function effectively as CIs and CTs. Between April 27 and May 5, the team undertook a curriculum design sprint by performing a needs assessment, determining relevant goals and objectives, and developing content. The initial four-day curriculum consisted of 13 hours of synchronous live web meetings and 7 hours of asynchronous, self-directed study. Educational content emphasized the principles of COVID-19 exposure, infectious period, isolation and quarantine guidelines and the importance of prevention and control interventions. A priority was equipping learners with skills in rapport building and health coaching through facilitated web-based small group skill development sessions. The training was piloted among 31 learners and subsequently expanded to an average weekly audience of 520 persons statewide starting May 7, reaching 7,499 unique enrollees by August 31. Capacity to scale and sustain the training program was afforded by the UCLA Extension Canvas learning management system. Repeated iteration of content and format was undertaken based on feedback from learners, facilitators, and public health and community-based partners. It is feasible to rapidly train and deploy a large workforce to perform CI and CT. Interactive skills-based training with opportunity for practice and feedback are essential to develop independent, high-performing CIs and CTs. Rigorous evaluation will continue to monitor quality measures to improve the training experience and outcomes.

## Introduction

In March-April 2020, California was experiencing a rapid increase in COVID-19 incidence, and the State's Department of Public of Health needed to rapidly develop a coordinated pandemic response that included case investigation (CI) and contact tracing (CT). CI and CT, in combination with isolation of cases and quarantining and testing of contacts, are key elements of the COVID-19 response (1–6). CI and CT have been a cornerstone of the public health response to other infectious disease outbreaks for decades; as evidence of this, they have been used in various countries for HIV (7), Ebola (8), tuberculosis (9, 10), and hepatitis C (11) outbreaks. Moreover, the observation that countries with robust and expansive CI and CT programs have effectively controlled transmission of SARS-CoV-2 (e.g., Singapore and New Zealand) (12, 13) has led to calls for expansion of similar programs in the United States. Despite recognition that a large workforce was needed, few jurisdictions in the U.S., including California, had a comprehensive CI and CT training plan, a clear assessment of ongoing training needs, a plan to operationalize training on a large scale or adequate funds budgeted for training. Stay-at-home orders and widespread shifts to working remotely during the COVID-19 pandemic introduced additional challenges to training, which is typically conducted in-person.

We describe the experience in California, where a unique partnership between the University of California, San Francisco (UCSF), University of California, Los Angeles (UCLA) and the California Department of Public Health (CDPH) rapidly implemented a training program for CI and CT. The description outlined below highlights three mutually related facets that illustrate the uniqueness of the effort, but also provide lessons for other jurisdictions. Firstly, the training program was designed and implemented to be delivered rapidly and at scale. The state's target of training several thousand contact tracers and case investigators by mid-summer demanded an approach to training that leveraged online learning resources. Secondly, the training program design was informed by a conviction that asynchronous *didactic* learning would be insufficient to prepare a workforce to effectively contain COVID-19. The initial audience for this training was redirected civil servants, such as census workers and librarians, who had very limited public health experience. As a consequence, the training needed to focus both on theory and the practice of CI and CT (14). As we highlight, the curriculum was distinct from other available CI/CT training tools that focused primarily on teaching principles without also providing training on the practical skills critical to reaching those impacted by COVID-19. Thirdly, the training program was developed with early recognition that learners needed not only the knowledge and skills to perform this essential function, but also sensitivity and empathy to reach those communities most impacted by COVID-19. Accordingly, we embedded a module in the core training focusing on cultural humility and the social context of people's lives (15, 16). The main objective of this paper is to highlight the different components of the curriculum development process and implementation, so that our approach can serve as a model of evidence-informed COVID-19 specific CI/CT instructional design.

## Methods and Pedagogical Framework

To guide the design and implementation of the project, a Steering Committee was formed consisting of 15 infectious disease epidemiologists, clinicians, community health scientists, instructional designers, learning specialists and evaluation experts from UCSF, UCLA, and CDPH. This group had expertise in professional training of both a public health workforce and others who are not public health professionals. This group took a systemic approach to curriculum design as outlined below.

From April 27 to 30, 2020, the Steering Committee undertook a rapid asset and needs assessment to determine goals, objectives and strategies of the new training program. The rapid asset and needs assessment consisted of:

1. Identifying areas of expertise of the team. Several task forces were created to manage the many logistics of this large and comprehensive effort (Table 1).
2. Identifying existing training resources, both those available freely online and those previously created by the team members.
3. CDPH conducting a statewide survey of the 61 local health jurisdictions to assess the needs to support COVID-19 CI and CT programs and demand for a training program. This survey detailed assessment of each jurisdiction's CI/CT workforce, including the size of their workforce, the digital platform(s) used and the training program(s) used to equip people to do CI and/or CT.

**Table 1:** Virtual Training Academy (VTA) task forces.

**Task forces**	**Objectives/tasks**
Curriculum task force	• Ensure coordination across modules to maintain consistency in content • Incorporate updates to CDC and CA guidelines • Review evaluation data and CDPH feedback to support systematic update process • Coordinate planning and instructional design • Develop and update asynchronous content • Recommend appropriate division and scheduling of synchronous and asynchronous content
Registration task force	• Report weekly course data on enrollment, completion, demographics, etc. • Send records of completion • Update course registration landing page • Create courses and skill development labs in registration system and Zoom links in Canvas • Provide timely information for course marketing by CDPH • Problem solve access to LMS and Zoom-hosted activities
Facilitator task force	• Hire a workforce of Leads and facilitators to facilitate CI & CT skills labs • Create flexible schedule of facilitators and adapt week-to-week depending on student enrollment • Provide training to new facilitators and identify ongoing training needs
Production task force	• Ensure Leads and facilitators are scheduled and present at live webinars and skill development labs • Ensure readiness and timeliness of educational materials, incorporating revisions and relaying content to LMS team • Manage production of skill development labs, including breakout group assignments
Monitoring & evaluation task force	• Design, implement, and update pre- and post-course tests and surveys • Conduct key informant interviews with those who successfully completed the program • Provide continuous feedback to Curriculum Task Force based on results of surveys • Oversee manuscript writing and development

The needs assessment identified an urgent need for training—most jurisdictions had limited capacity to provide training—as well as a paucity of high-quality training materials focused on teaching COVID-19 CI and CT. Subsequently, between April 27 and May 5, 2020, the team rapidly developed a training curriculum that would enable learners from diverse backgrounds and experiences to quickly acquire the competencies necessary to function effectively in the roles of either a CI or a CT for the COVID-19 response. Based on results of the asset and needs assessment, the team first determined the learning objectives and modules of the pilot CT training (Table 2). The Curriculum Task Force then matched the content, learning activities and assessment measures to the learning objectives for each module. Content and learning activities, including asynchronous self-directed online study and live web meetings for presentations and skill development, were designed to provide multiple means for students to engage in the material to accommodate different learning styles (17). The Curriculum Task Force requested and received an external review of the curriculum and content from non-involved UCSF faculty and state public health partners with relevant subject matter expertise. Subsequently, by May 18, a complementary track for CI (Table 2) with associated skills sessions was developed and piloted, building on the existing CT modules.

**Table 2:** Modules and learning objectives of California Virtual Training Academy (VTA) case investigation and contact tracing, August 2020.

**Module name**	**Learning objectives**
Module 1. Basic epidemiology of COVID-19	1. Define SARS CoV-2, “novel coronavirus” and COVID-19. 2. List the most common symptoms of COVID-19. 3. Describe the incubation and infectious periods of COVID-19. 4. Describe how to prevent COVID-19 transmission. 5. Describe the current geographic and demographic distribution of COVID-19.
Module 2. Overview of CI & CT	1. Summarize the goals and activities of CI and CT. 2. Explain the relationship between CI and CT. 3. Identify when to refer individuals or situations to medical, social or supervisory resources.
Module 3. Comprehensive containment strategies	1. Describe the purpose of isolation. 2. Describe the purpose of quarantine. 3. Calculating dates of isolation and quarantine periods. 4. Identify the dates that a case's isolation period should begin and end. 5. Identify the dates that a contact's quarantine period should begin and end. 6. Understand wraparound services and how to develop a plan for a successful quarantine or isolation.
Module 4. Case Investigation Basics for COVID-19	1. Describe the case investigator roles and responsibilities. 2. Learn how to determine the contact elicitation window during a case investigation interview. 3. Identify when to refer individuals or situations to medical, social or supervisory resources.
Module 5. How to conduct CT Interviews	1. List the materials needed to conduct a CT interview. 2. Discuss and address contact concerns about being notified or interviewed. 3. Summarize the sequence of activities involved in conducting a CT interview. 4. Explain the rationale behind each section included in the CT interview questionnaire.
Module 6. Respecting the context: cultural humility matters	1. Explain implicit bias and how it might impact a CI or CT interview. 2. Define cultural humility and describe how it relates to CI and CT.
Module 7. The CI Interview	1. List the eight key components of the CI interview. 2. Explain why it is important to ask questions related to health history. 3. Explain why it is important to ask questions about symptoms and where someone with COVID-19 spent time leading up to the start of their symptoms.
Module 8. Starting the conversation: interviewing skills and setting the agenda	1. Define active listening skills to apply to interviews. 2. Learn the steps to setting the agenda to begin interview process.
Module 9. Building on case/contacts' knowledge & motivation: health coaching tools	1. Describe why it is necessary to actively engage case/contacts in a CI/CT interview. 2. Use Ask-Tell-Ask and Closing the Loop techniques to reinforce knowledge about COVID-19 and prevention strategies during an interview. 3. Demonstrate use of Action Planning to support successful quarantine planning. 4. Successfully conduct the CI/CT phone script in a variety of case/contact scenarios using cultural humility, Ask-Tell-Ask, Closing the Loop, and Action Planning.

## Learning Environment

### Course Platforms

The initial four-day CT curriculum consisted of approximately 13 h of synchronous live web meetings and 7 h of asynchronous, self-directed online study (Table 3). Asynchronous self-study consisted of materials easier to review according to learners' schedules, such as videos, job aids and the Association of State and Territorial Health Officials (ASTHO) online-only course, “Making Contact: A Training for COVID-19 Contact Tracers” (https://www.astho.org/COVID-19/Making-Contact-Tracer-Training/). Asynchronous learning content was hosted on UCLA Extension Canvas learning management system (https://community.canvaslms.com/t5/Canvas-Basics-Guide/What-is-Canvas/ta-p/45). Zoom web conferencing platform was used to teach synchronous content including webinars and skill development laboratories (https://zoom.us/).

**Table 3:** Agenda of the California Virtual Training Academy course in Case Investigation and Contact Tracing, August 2020.

Orientation Day (Monday)	Attend Zoom Webinar 9:00 AM – 10:45 AM • 9:00 AM – 9:15 AM Course Introduction • 9:15 AM – 10:00 AM Introduction to Canvas, Skill Development Lab Registration, and Zoom • 10:00 AM −10:05 AM BREAK • 10:05 AM – 10:45 AM CDPH Orientation - Logistics (for state employees only) • Complete Required Self-Study Activities 1. Register for Skill Development Labs. 2. Complete the Self-Study Activities before Day 1: • ASTHO: The Basics of Coronavirus Disease 2019 *(approx. 45 min.)* • ASTHO: The Basics of Contact Tracing *(approx. 45 min.)* • ASTHO: Case Monitoring and Resources *(approx. 30 min.)* • Pre-training Self-assessment (due by 9am on Day 1) • Pre-training Knowledge Check (due by 9am on Day 1)
Day 1 (Tuesday)	Attend Zoom Webinar 9:00 AM – 12:15 PM • 9:00 AM – 9:20 AM Canvas Recap • 9:20 AM – 9:50 AM Introduction to Public Health, Epidemiology, and COVID-19 • 9:50 AM −10:30 AM Overview of Contact Tracing • 10:30 AM – 10:40 AM BREAK • 10:40 AM – 11:20 AM Comprehensive Containment Strategies • 11:20 AM – 11:30 AM Video Words from current CI + CTs • 11:30 AM – 11:45 AM BREAK • 11:45 AM – 12:15 PM Case Investigation Basics for COVID-19 • Complete Required Self-Study Activities 1. Register for your Skill Development Labs. 2. Complete the Self-Study activities before Day 2: • ASTHO: Effective Communication and Interviews *(approx. 1 hr)* • Confidentiality video *(approx. 10 min.)* • Contact tracing interview demo *(approx. 13 min.)*
Day 2 (Wednesday)	Attend Zoom Webinar 9:00 AM – 11:15 AM • 9:00 AM – 9:30 AM How to Conduct a Contact Tracing Interview • 9:30 AM – 10:15 AM Respecting Context • 10:15 AM – 10:30 AM BREAK • 10:30 AM – 11:15 AM Case Investigation Interview • Attend Skill Development Labs: Part 1 Participate in two 90-minute Skill Development Labs covering both Case Investigator and Contact Tracer skills. • 1:00 – 4:15 • Case Investigator Interview: Part 1 – Introduction (90 min), and • Contact Tracer Interview: Practice Script (90 min) • Download Learner Guide & Practice Script for use during Skill Development Lab Canvas Course.
Day 3 (Thursday)	Attend Zoom Webinar 9:00 AM – 10:35 AM • 9:00 AM – 9:45 AM Starting the Conversation • 9:45 AM – 9:50 AM BREAK • 9:50 AM – 10:35 AM Health Coaching • Attend Skill Development Labs: Part 2 • Participate in two 90-minute Skill Development Labs (use same zoom link) covering both Case Investigator and Contact Tracer skills. • 1:00 – 4:15 • Case Investigator Interview: Part 2 – Contact Elicitation (90 min), and • Contact Tracer Interview: Interview & Health Coaching Skills (90 min) • Download Learner Guide & Practice Script for use during Skill Development Lab Canvas Course. • Complete Required Self-Study Activities 1. CalCONNECT Platform Interview Demo Video *(approx. 30 min.)*
Day 4 (Friday)	Attend Zoom Webinar 9:00 AM – 11:00 AM • 9:00 AM – 9:05 AM Announcements • 9:05 AM – 9:20 AM CalCONNECT Platform Introduction • 9:20 AM – 10:10 AM VTA Rapid Review • 10:10 AM – 10:20 AM BREAK • 10:20 AM – 10:55 AM Panel Discussion with Existing Contact Tracers • 10:55 AM – 11:00 AM Conclusion • Attend OPTIONAL Skill Development Labs • Topics: • CT Day 2 Additional Practice • CT Day 3 Additional Practice • Case Investigation Interview Practice • Spanish Practice Lab • This Spanish skill development lab is open for all levels of Spanish speakers Complete Required Self-Study Activities 1. Complete the Final Course Assessments: • Post-training Self-assessment • Post-training Knowledge Check • Final Course Participation Survey • STATE WORKERS ONLY: Information Privacy and Security Training Acknowledgment

### Webinars

With an initial plan to train 2,500 learners per week, instructor-led presentations were delivered using the Zoom video webinar function, which enables up to 3,000 participants to join simultaneously (including learners, observers and facilitators). These webinars addressed much of the core content, delivered in a didactic “presentation format” by module presenters, who were Steering Committee members who had expertise in the topic being presented. Module presenters were encouraged to make the webinars as interactive as possible using four techniques: (1) poll questions using the Zoom poll function; (2) live audience response using the Zoom chat function (speaker posed question and quickly acknowledged themes and notable responses gathered from learners in the chat); (3) real-time texting using the Zoom Q&A function (supporting faculty fielded written Q&A during the presentation and curated questions for live Q&A discussion); and (4) live Q&A discussion with speaker at end of each presentation.

### Skill Development Labs

In addition to knowledge delivered through didactic presentations, the team developed a curriculum emphasizing learning practical interviewing skills through four 1.5-h “skill development labs.” Skill sessions covered (1) role-play practice using a CT script, (2) applying interviewing and health coaching principles to challenging situations, and (3) two CI-track skill sessions for role-play focused on challenging segments of the CI script (see Supplementary Material). Skill development labs were led by a lead facilitator, who introduced role-play activities to the whole group, after which learners were separated into small breakout groups of 5–10 learners. Trained facilitators were typically assigned to each breakout group, although in some cases when enrollment was high, the facilitators rotated between breakout groups. During the small breakout groups, learners practiced delivering the CI or CT script using the interviewing skills and health coaching techniques described during the webinars. For example, in the first CT skill development lab, learners practiced reciting the introduction of the CT interview script, conducting an interview with a contact and addressing common concerns a contact might have about being notified or interviewed. The facilitators' role included leading the small group in introducing themselves, providing instructions for each activity, ensuring each learner had an opportunity to practice, providing tailored, in-depth feedback to each learner to strengthen their skills, keeping track of the time and answering learner questions. The opportunity to practice interviewing skills is what distinguished the VTA courses from other available online CI and CT courses.

Skill development labs met on Days 2 and 3 of the 4-day course using the Zoom meeting function. CT learners were required to participate in two labs, and CI learners were required to participate in four labs. Because the Zoom meeting function allowed a maximum of 300 participants, we limited the number of learners in each skill development lab to 250, which allowed for 10–50 facilitators and production staff to join as well (Table 4). With a maximum of 2,500 learners joining the course per week, ten daily skill development labs were required to accommodate all learners. On Day 4, we offered optional skill development labs where learners could experience extra practice or make up a skill development lab missed during the week.

**Table 4:** Structure of daily skill development labs of the California Virtual Training Academy (VTA) course in Case Investigation and Contact Tracing.

	**CT Skills lab 1**	**CT Skills lab 2**	**CT Skills lab 3**	**CT Skills lab 4**	**CI Skills lab 5**
11:00–12:30	2 lead facilitators 25 facilitators 250 learners	2 lead facilitators 25 facilitators 250 learners	2 lead facilitators 25 facilitators 250 learners	2 lead facilitators 25 facilitators 250 learners	N/A
12:45–2:15	2 lead facilitators 25 facilitators 250 learners	2 lead facilitators 25 facilitators 250 learners	2 lead facilitators 25 facilitators 250 learners	2 lead facilitators 25 facilitators 250 learners	2 lead facilitators 25 facilitators 250 learners
2:30–4:00	2 lead facilitators 25 facilitators 250 learners	2 lead facilitators 25 facilitators 250 learners	2 lead facilitators 25 facilitators 250 learners	2 lead facilitators 25 facilitators 250 learners	2 lead facilitators 25 facilitators 250 learners
TOTAL	3,000 CT learners				500 CI learners

### VTA Facilitators and Production Staff

Given the large number of learners each week and the need to engage them in small group practice sessions, the VTA required a large number of trained facilitators. Facilitators were recruited rapidly among public health graduate students, staff and faculty of UCLA and UCSF, as well as retired health workers. A total of 104 facilitators were initially recruited and trained for 4 h over two consecutive weeks in May 2020. Training consisted of an overview of the CI and CT courses, navigating the Canvas platform and Zoom, review of facilitator roles, content of the skill development labs, and best practices for facilitating small groups. The training was conducted on Zoom, which also allowed new facilitators to practice some of the Zoom functions. Facilitators worked for the VTA part-time between 2 and 15 h per week. As new facilitators joined the VTA, they attended an onboarding meeting on role and logistics, participated in the CI and CT training, and then shadowed experienced facilitators during a skill development lab before facilitating on their own. All facilitators met during a bi-weekly “facilitator check-in” consisting of an overview of the number of learners who had completed the course, announcements and updates to learning materials, and ongoing training and development. All activities of the VTA facilitators were coordinated by the Facilitator Task Force consisting of nine members who met twice weekly to coordinate the activities described above.

In addition to facilitators, implementing the VTA required production and technical support staff. Production staff managed the Zoom webinars and meetings by setting up waiting rooms for participants, recording the presentations, sharing slides and videos, enabling or disabling the chat function, facilitating poll questions and managing breakout rooms, among other duties. Production staff were primarily college students hired on a part-time basis. Technical support was provided by UCSF Educational Technology Services and UCLA Extension and included helping learners and staff with technical problems (such as audio and video) and answering technical questions using the Q&A function of Zoom. The Production Task Force hired and managed the production and technical support staff, in addition to other duties, ensuring the VTA courses ran smoothly each week. Production and technical support were crucial components in taking the VTA training courses to scale.

### Selection of Learners

The VTA training courses were offered to the following groups of people performing or training to perform contact tracing or case investigation activities: (1) California state employees re-directed to COVID-19 work; (2) local health department employees, contractors and partners designated to provide support to local health department COVID-19 contact tracing activities; (3) tribal nations. Because of the high demand, the courses were not open to the general public in order to save space for learners who would be doing case investigation and contact tracing activities. Learner registration and enrollment was managed by the Registration Task Force and UCLA Extension using Destiny, a registration portal system. Eligibility for each person enrolled in a course was verified by VTA staff before access to the course was given.

### Course Evaluation

A program evaluation plan, aligned with the course goals and objectives, was designed to capture learners' sociodemographic characteristics, course participation, knowledge and self-assessment of skills. Learners who passed the knowledge assessment with ≥ 70% correct were emailed a record of completion within 2 weeks of course completion. Learners provided feedback on the course content and delivery at the time of training and again 2 weeks after completion of the training. Feedback was used to inform iterative improvements of the course content and delivery and logistical changes to improve learner experience.

## Results

### Pilots and Move to Canvas Platform

The VTA training program was piloted in early May 2020 to two groups of learners affiliated with the San Francisco Department of Public Health (*n* = 31) before being rolled out to a statewide audience. Feedback was collected from these learners through a Qualtrics survey; learners wanted less didactic training and more time for practicing interviewing skills. Incorporating feedback from the pilot, we implemented a second and expanded CT training to a group of 547 learners on May 7–13, adding more time for small group skill development labs. From May 18 onwards, the CI and CT courses were delivered on the UCLA Canvas platform; the first cohort to use Canvas consisted of 443 learners.

### Sociodemographic Characteristics of Learners

An average of 520 learners a week were trained (range 163–1,120) during the first 16 weeks of training (May 7–August 31, 2020) (Table 5). Most learners held a non-public-health-related job title, such as librarian or city attorney, before being redirected to COVID-19 work (71.9%). Responses from self-reported demographic questionnaires indicated that the majority of learners were female (71.3%), and many were college graduates or had more advanced degrees (68.3%), were White/Caucasian (48.2%), and not of Hispanic, Latinx or Spanish origin (69.7%). The age distribution of learners was spread evenly across age groups from 25–64 years, with a smaller proportion of learners aged 18–24 (7.6%) and 65 or older (8.0%). Spanish (14.8%) was the most frequently spoken language in addition to English (63.4%), with 18 other languages selected by at least one learner. At the start of each week, the Monitoring and Evaluation Task Force analyzed and distributed the demographic data of the learners to VTA staff in order to help presenters and facilitators better prepare for incoming cohorts.

**Table 5:** Demographics of training staff and learners registered in the California Virtual Training Academy (VTA), May-August 2020.

	**Learners number (%)**
Survey Responses	6,342
**Gender identity**	5,934
Female	4,231 (71.3)
Male	1,869 (31.5)
Transgender male/transman	2 (0.0)
Transgender female/transwoman	2 (0.0)
Genderqueer	15 (0.0)
Non-binary/third gender	1 (0.0)
Other	3 (0.0)
Decline to state or missing	218 (3.7)
**Age group**	5,795
18–24	440 (7.6)
25–34	1,136 (19.6)
35–44	1,062 (18.3)
45–54	1,211 (20.9)
55–64	1,146 (19.8)
65+	466 (8.0)
Decline to state or missing	334 (5.8)
**Highest level of education achieved**	5,795
High school grad/GED or equivalent	376 (6.5)
Some College or Associate's Degree	1,407 (24.3)
College Graduate	2,186 (37.7)
Some Graduate School	287 (5.0)
Graduate, clinical, or professional degree	1,481 (25.6)
Other	21 (0.4)
Missing	37 (0.6)
**Pre-COVID-19 job title**	**5,183**
Non-public health related (librarian, city attorney, etc.)	3,726 (71.9)
Other allied health professional (health educator, epidemiologist, etc.)	1,074 (20.7)
Disease investigator	57 (1.1)
Public health administrator	101 (2.0)
Public health nurse	222 (4.3)
Missing	3 (0.1)
Racial background^*^	5,840
American Indian or Alaska Native	144 (2.5)
Asian	1,039 (17.8)
Black or African American	551 (9.4)
Native Hawaiian or Pacific Islander	94 (1.6)
White/Caucasian	2,812 (48.2)
Other	40 (0.7)
Decline to state or missing	1,160 (19.9)
**Hispanic, latinx, or spanish origin**	5,730
Yes	1,347 (23.5)
No	3,994 (69.7)
Decline to state or missing	389 (6.8)
TOTAL	5,730 (100.0)
Language spoken fluently^*^	7,291
English	4,620 (63.4)
Spanish	1,077 (14.8)
Hindi	112 (1.5)
Vietnamese	110 (1.5)
Tagalog	106 (1.5)
Mandarin	103 (1.4)
Cantonese	94 (1.3)
Farsi	75 (1.0)
Punjabi	58 (0.8)
Arabic	33 (0.5)
Other^**^	688 (9.4)
Missing	215 (2.9)

*N/A, Data not available*.

* *Questions where learners were asked to “select all that apply”*.

** *Korean, Chinese, Cambodian, Japanese, Hmong, Thai, Laotian, Russian, Armenian, Other not named*.

*Percentages may not add up to 100.0% due to rounding. Answer choices with 0 responses are not represented in this table. All learners may not be included here as responses were not required to answer and surveys changed in the first four cohorts*.

### Iteration and Development of New Content

The VTA continued to redesign the course content and format based on feedback from learners and facilitators. Module presenters were invited to make updates to the slides or materials from week to week based on updated guidelines and evaluation results (such as knowledge scores on specific module-related questions). Substantial revisions of the course content were made in July 2020 and were based on learner and external CDPH observer feedback. Revisions included expansion of cultural humility content (see Supplementary Table 1), improved skill development activities and scripts, additional skill development labs (optional practice and development of Spanish-speaking practice labs and materials), a reduction in live didactic time with concomitant increase in self-study video options, and expanded use of real-life stories from CIs and CTs in the field (live Q&A and video testimonies). In August 2020, in response to increased demand for case investigator training, the VTA created a new CI course for alumni of the VTA CT course that allowed these learners to attend only CI webinar modules and skill development labs, but not repeat content they had previously covered.

## Discussion

To our knowledge, this is the first time that a COVID-19 contact tracing skills-based training program has been adopted at scale in the U.S. The program is unique given both its focus on equipping learners with the interviewing and health coaching skills necessary to perform this essential task and the rapidity with which it was implemented. The iterative approach to curriculum development and the emphasis on cultural humility are also central tenets of the California COVID-19 contact tracing training effort. We assert that the California contact tracing program offers not only a model in *how* to scale COVID-19 contact tracing training, but also *what* core components should be included in such a training program. Outlined below are several reasons why the California CI and CT training program offers a model for other jurisdictions:

Firstly, the CI and CT training program, targeted to learners with limited public health experience, was implemented with considerable speed. Within 16 weeks of inception, the program had trained 7,499 unique learners who could then be deployed as part of the state's COVID-19 public health response workforce. This rapid scale-up was achieved through a unique partnership between CDPH and the University of California, leveraging CDPH's ability to quickly mobilize learners from across the state and the university's capacity to quickly implement an online training program. While the need for social distancing precluded in-person training, it offered a unique opportunity to use the UC's existing online educational capability. Moreover, using an online format allowed the training team to reach learners from across the large state of California.

Secondly, a key facet of the California CI and CT training program is its central emphasis on equipping learners with critical practical skills necessary to perform case investigation and contact tracing effectively. While other available contact tracing training curricula highlight the importance of developing interviewing skills, few provide the infrastructure to support learners to develop these skills in live, interactive web-meetings. Given many of the learners undertaking contact tracing in California had no public health experience, we assert that this ‘hands-on approach' to training was critical. Moreover, this approach is supported by data from contact tracing training programs for other diseases including syphilis and tuberculosis, where skills-based modules are essential to enhancing the proficiency of learners (18, 19). The training also included modules on health coaching skills. While health coaching is recognized primarily as a method to improve chronic disease outcomes, we adapted its core skills to equip learners with basic skills to establish a collaborative relationship with contacts and cases, to assess and build on contact knowledge of COVID-19 prevention strategies, and to explore and address ambivalence around recommendations such as quarantine and isolation.

Implementing “hands-on” skill development labs for up to 2,500 learners a week presented logistic challenges and required a large workforce of more than 100 facilitators to ensure that breakout groups were small enough to allow effective skill development. Nonetheless, the skill development labs afforded opportunities for practice and feedback necessary to the development of independent, high-performing contact tracers and case investigators. Further research is ongoing to understand better the extent to which these practical sessions affected learners' confidence and competency and therefore improved public health.

Thirdly, the description of California's contact tracing training program also highlights the importance of ensuring that the COVID-19 CI and CT workforce appreciate diversity, inclusion, and cultural humility as central tenets of the public health response. Substantial research has already documented the way that COVID-19 has disproportionately affected specific ethnic and racial communities in the U.S. (20–22). The profound deleterious impact of structural racism on the COVID-19 public health response is also well described (23, 24). In concert with strategies to increase COVID-19 CI and CT workforce diversity, optimize minority community engagement in CI and CT efforts and target the social determinants of health (25), we assert that the training program highlighted here can play an important role in addressing the legacy of health inequities in the U.S.

### Further Extensions and Expansions of the VTA

Beyond CI and CT training, the VTA team is now leveraging the training expertise and the educational infrastructure to respond to other COVID-19 public health training needs and to enhance the existing content in response to the evolving epidemic in California. For example, to address an increasing demand for outbreak investigators in local health jurisdictions, the VTA team developed and implemented an outbreak management course. This was piloted for 38 learners on August 25–27, 2020, and was subsequently incorporated into the standard VTA course options. Given the huge impact that COVID-19 is having in Latinx communities in California, a weekly optional skill development lab in Spanish, with Spanish-speaking facilitators and translated materials, was added in early August 2020. This lab equips CIs and CTs to more adeptly reach the Latinx community. Content on respecting context and cultural humility was expanded to encourage learners to explore implicit bias and how it might affect the CI and CT interview. Learners were encouraged to examine their own beliefs, cultural identities and values in order to recognize and challenge power imbalances and better address the needs of cases and contacts. In addition, to support continuous learning in the ever-changing world of COVID-19, the VTA has created several “communities of practice” for groups of CIs and CTs in local health jurisdictions, as well as a group for state redirected employees to meet online weekly to create community and share experiences, updates and best practices. Finally, the VTA developed school-specific initiatives to support schools' reopening, including technical assistance to local health jurisdictions and risk communication training.

## Acknowledgment of Constraints

A limitation of the existing training program is the limited breadth of topics covered within the training curriculum. Notably, training to date has not included content on mental health and/or the importance of trauma-informed care. As the epidemic in California has unfolded, it has become increasingly apparent that knowledge of these domains is critical for CIs and CTs and thus they need to be incorporated into the curriculum. Furthermore, the VTA offered a general training in CI and CT and did not offer specific training on the standard operating procedures of each local health jurisdiction. Therefore, local health jurisdictions needed to offer specific training and onboarding for their CIs and CTs. Finally, as more was learned about COVID-19 and VTA trainings were updated with current information, there was not an easy way to communicate these updates to earlier cohorts. Again, this fell to local health jurisdictions to keep their local workforces updated. Some of these issues may be addressed through ongoing in-service training opportunities, combined with the development of local communities of practice for CIs and CTs.

## Conclusions

CI and CT have been used for multiple diseases to contain outbreaks (7–11) and are now at the core of COVID-19 containment activities. A robust and resilient response to COVID-19 depends on local and state public health departments having the capacity to develop and maintain CI and CT capability. Time is of the essence given the need for workforce training. The lessons from California can inform how other states respond. The VTA curriculum was designed to equip CIs and CTs with essential skills to provide culturally sensitive care by incorporating interactive learning strategies focused on cultural humility, rapport-building and health coaching techniques (15, 16).

## Data Availability Statement

The original contributions presented in the study are included in the article/Supplementary Files, further inquiries can be directed to the corresponding author/s.

## Author Contributions

All authors served on the VTA Steering Committee and/or task forces. DB, AA, LC, ADi, ADo, AG, AK, AM, HM, PM, MP, SS, KW, RW-G, and MR determined content of the VTA courses, wrote or revised training goals and objectives, selected educational or evaluation strategies, implemented the curriculum, and contributed to revisions of the manuscript. MF, EA, CD, MG, AK, AP, MP, and SS collected, managed or analyzed learner data. DB, MF, and MR drafted and revised the manuscript. All authors read, provided comments to, and approved the final manuscript.

## Conflict of Interest

The authors declare that the research was conducted in the absence of any commercial or financial relationships that could be construed as a potential conflict of interest.

## Publisher's Note

All claims expressed in this article are solely those of the authors and do not necessarily represent those of their affiliated organizations, or those of the publisher, the editors and the reviewers. Any product that may be evaluated in this article, or claim that may be made by its manufacturer, is not guaranteed or endorsed by the publisher.
