# Supplementary material for: California's COVID-19 Virtual Training Academy: Rapid Scale-Up of a Statewide Contact Tracing and Case Investigation Workforce Training Program
Source: Front Public Health. 2021 Aug 9;9:706697. doi: 10.3389/fpubh.2021.706697 (PMC8381767; doi:10.3389/fpubh.2021.706697)
Supplement: Supplementary file 1 [file Table_1.DOCX]

Supplemental Table 1. Cultural humility content of California Virtual Training Academy (VTA) case investigation and contact tracing course, August 2020

| **Module 4. Respecting the Context (Cultural Humility Matters)^1^** | |
| --- | --- |
| Learning Objectives | By the end of the session, the learner will be able to:   1. Explain implicit bias and how it might impact a contact tracing or case investigation interview 2. Define cultural humility and describe how it relates to contact tracing and case investigation |
| Topics | - What is bias? (conscious and unconscious)   - Definition of unconscious bias   - Why we have biases   - How perception influences biases   - The evidence about unconscious bias   - Key characteristics of unconscious bias - Challenging implicit bias |
|  | - What is cultural humility?   - A practical definition of culture   - Cultural humility principles   - Recognize and challenge power imbalance   - Institutional accountability   - Cultural humility approach |
|  | - Example in case investigation and contact tracing script: Sensitive personal information gathering   - Sexual Orientation & Gender Identity (SOGI)   - Why is asking about SOGI important?   - How to frame and how to ask the questions   - Critical self-awareness |
|  | - Guidelines for cross cultural interactions: Empathy, curiosity and respect |

1. *To request course materials, please contact* [*Debbie.bainbrickley@ucsf.edu*](mailto:Debbie.bainbrickley@ucsf.edu)
