# Supplementary material for: California's COVID-19 Virtual Training Academy: Rapid Scale-Up of a Statewide Contact Tracing and Case Investigation Workforce Training Program
Source: Front Public Health. 2021 Aug 9;9:706697. doi: 10.3389/fpubh.2021.706697 (PMC8381767; doi:10.3389/fpubh.2021.706697)
Supplement: Supplementary file 2 [file Data_Sheet_1.PDF]

Supplemental Material

Learner Guides for the California Virtual Training Academy (VTA) case investigation and contact tracing course, August 2020

Notes:

1. Learners attend live web-based skill development sessions on Zoom. They are separated into small groups of 5-6 learners and one facilitator per Zoom breakout room to participate in skill development activities, using the learner guides and practice scripts for case investigators and contact tracers.
2. To request course materials, please contact Debbie.bainbrickley@ucsf.edu

How to Conduct a Case Investigation Interview –

Part 1: The Introduction

DAY 2 Skills Lab Learner Guide

PURPOSE

To provide case investigators with an opportunity to practice the **Introduction sections** of the Case Investigation Interview script.

INTRODUCTIONS

Small Group Breakout Practice:

1. Keep video cameras on when possible. Start with a brief round of introductions providing name and where you work.
2. Quickly decide who will start the first role play as **CASE INVESTIGATOR** and **COVID-19 CASE** and who will serve as **MONITOR** (time-keeper), then begin.
3. After each role play + debrief, switch roles until everyone has had a chance to practice the Case Investigator role. Continue a second round of practice if/as time allows.

CASE INVESTIGATOR ROLE – You are calling a person with a new positive COVID-19 test result (the case). Use the Case Investigation script on pages 2-4 of this handout to guide you.

COVID-19 CASE ROLE – You are playing the person with a new positive COVID-19 test result (the case). Respond to questions using a scenario that feels comfortable for you, drawing from your life experiences. Vary the scenario from those played by others going before you.

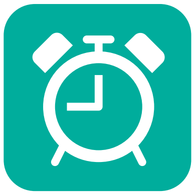

Quick Introductions:
Your name + work site

Role play: 7 minutes

Debrief: 2-3 minutes

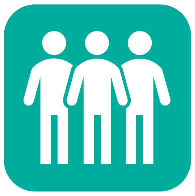

Group size: 3-5 people

MONITOR – Your role is to keep the session on track. Allow the **role play to be conducted for 7 min max** or until they reach the end of the script (whichever comes first), then stop and ask the group to debrief for 2-3 min.

OBSERVERS- everyone else in the small group not assigned as **Monitor**, **Case Investigator**, or **COVID-19 Case**. As you observe the role play, look/listen for and take note of the following, and be prepared to share your observations (as time allows) during the debrief:

- ✓ Were all sections of the script covered? (e.g., confirming identity, confidentiality)
- ✓ What things were done well? (i.e. asked open-ended questions, established rapport)
- ✓ What areas have potential for improvement or were missed?

COVID-19 Case Investigation Interview Script (PART 1)

CASE INVESTIGATOR ROLE: As the Case Investigator, use the sample script below to guide you through an interview with a person who has recently tested positive for COVID-19. Today's date is the date the test result was reported (the test obtained 2 days prior). You are calling from your work setting and walking through just the Call Introduction, COVID-19 Information and Call Objectives sections of the case interview.

A. Call Introduction

[The full script has guidance on situations including if person you are calling has died, is physically unable to speak, or is a minor.]

Hello, my name is **[Your Name]** and I am calling from the **[jurisdiction]** Department of Public Health regarding an urgent health matter.

Can I confirm that I am speaking with **[Name of Case]**? *If no → ask to speak to that person*

If yes → Thank you. To ensure I am speaking with the correct person and protecting confidentiality, I need to ask you to confirm your date of birth and home address. May I have your birthdate or just the month and date, please?

And your current address?

Thank you for confirming this information.

I am with the Department of Public Health, calling in regard to your COVID-19 test result. It's important to me that you understand that **everything you and I talk about is confidential**. What that means is that I will only share the information we discuss with other public health workers who are directly involved with your care. Are you in a place where you can speak privately?

Have you received your test result yet?

If yes → [Ask what information was provided and by whom]

[In some cases, the patient may not have received their results from their primary care provider prior to the investigation call. Therefore, the case investigator will be the first person to notify them that they are COVID positive. Proceed slowly, and with empathy, as this news may be unsettling.]

If no → As you know, you were recently tested for COVID-19. I'm calling to let you know your test result has come back positive, meaning that you are infected with the coronavirus and have COVID-19.

Covid-19 is a reportable infectious disease which is why the public health department has received your test result. We are calling everyone who has tested positive in the state to ensure they have the information they need to keep safe and to prevent further spread of the virus.

How are you feeling today?

Do you have time to speak with me now?

If yes → Continue.

If it is not a good time to speak right now, ask→ When is a better time to call?

Is this the best number to get a hold of you at? How else can we reach you?

B. COVID-19 Information

What do you know about COVID-19?

[Tailor the information below to respond to the person's questions and provide fact-based information about COVID-19.]

COVID-19 is **extremely contagious**, and it spreads very easily through contact.

COVID-19 is **caused by a new virus** and exists in almost every country.

The virus can cause many types of symptoms but **mostly affects the respiratory system, causing fever and cough.**

It can spread from an infected person to others when they cough, breathe, sneeze or touch a surface that other people then touch.

Luckily, most cases are mild or moderate and **most people don't need to be in the hospital. Some cases can get more serious** and cause pneumonia and breathing difficulties.

Since I'm not involved with your medical care, I cannot provide you with medical advice. If you need additional medical information, please contact your healthcare provider.

[Based on response to earlier question about whether test result had been received, if they already spoke with their medical provider about their test result, proceed to Call Objectives].

Do you have a regular healthcare provider?

If yes, → continue to Call Objectives

If no, → To assess your symptoms and risks you should speak with a clinician. *[Refer to LHJ guidance regarding referrals.]* If ok with you, I will refer you to *[community clinic and/or resource coordinator]* who can try to help set you up with a medical provider that is accepting new patients. If neither of these options work for you, you can go to an urgent care or the emergency room if you are not getting better or you feel like you are getting worse.

If the person brings up clinical questions or concerns: I am not able to answer any clinical questions you have; however, I could reach out to our team clinician. If you'd like, I could see if our clinician is available to call you back after we finish our call. Importantly, if you believe you are having a medical emergency, you should call 911. Some warning signs that you should go to the emergency room for are: trouble breathing, bluish lips or face, persistent pain or pressure in the chest, or new confusion or inability to arouse *(wake up)*, but there are other symptoms too. Otherwise, you should contact your healthcare provider.

[If there is any concern or questions, please refer to your supervisor.]

C. Call Objectives

I am calling today to provide you and those in your household with some guidance and support. To do this, I need to talk with you about two important items.

First, if you are living at home or with others, it is critical that you self-isolate immediately in order to protect your friends, family or other people you live with, so that nobody else becomes infected. You should use a separate room and separate bathroom if possible. We'll go over the home isolation instructions during this call to make sure we address any questions you may have.

Second, we will need to notify the people you have been in close contact with and let them know that they should be medically evaluated. Your name will not be shared with the people we call. I will talk more about this in a little bit.

[CONTINUE TO SET AGENDA FOR THE CALL]

- *Ask if there are any immediate concerns or questions to address during the call.*
- *Provide time estimate for the call and ask if ok to proceed.*

STOP ROLE PLAY AND DEBRIEF

How to Conduct a Case Investigation Interview –

Part 2: Contact Elicitation

DAY 3 Skills Lab Learner Guide

PURPOSE

To provide case investigators with an opportunity to practice the COVID-19 Case Investigation Interview sections pertaining to **eliciting information about close contacts**.

INTRODUCTIONS

Close contacts are those who spent ≥15 min within 6 feet of someone who tested positive for COVID-19, during the established contact elicitation window (symptomatic – 2 days before onset of symptoms through 1st day of isolation; asymptomatic – 2 days before the date the initial positive test was obtained through first day of isolation).

Small Group Breakout Practice:

4. Keep video cameras on when possible. Start with a brief round of introductions, providing name and where you work.
5. Quickly decide who will start in each role for the first role play (see role descriptions below) then begin.
6. After each role play + debrief, switch roles until everyone has had a chance to practice the Case Investigator role. Continue a second round of practice if/as time allows.

CASE INVESTIGATOR ROLE – You are calling a person with a new positive COVID-19 test result (the case). Use the Case Investigation script on pages 2-6 of this handout to guide you.

COVID-19 CASE ROLE – You are playing the person with a new positive COVID-19 test result (the case). Respond to questions the Case Investigator asks using a scenario that feels comfortable for you, drawing from your life experiences. Name several places you spent time during your infectious period and <u>2-3 close contacts</u>.

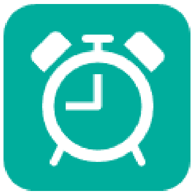

Quick Introductions:
Your name + work site

Role play: 10 minutes

Debrief: 2-3 minutes

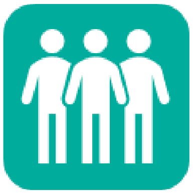

Group size: 3-5 people

MONITOR – Your role is to keep the session on track. Allow the **role play to be conducted for 10 min max** or until they reach the end of the script (whichever comes first), then stop and ask the group to debrief (2-3 min; case investigator to debrief first stating how the practice went).

OBSERVERS – everyone else in the small group not assigned as **Monitor**, **Case Investigator**, or **COVID-19 Case** will assume **Observer** role.

COVID-19 Case Investigation Interview Script (PART 2)

CASE INVESTIGATOR ROLE: Use the sample script below to guide you through an interview with a person who has recently tested positive for COVID-19. Today's date is the date the test result was reported (the test obtained 2 days prior). You are calling from your work setting and have completed the interview up to the point of discussing symptoms and contacts. This practice session covers just the sections of the script dealing with assessment for symptoms, establishing contact elicitation window, explanation of why you will be asking for information about contacts and asking questions to identify contacts at risk for COVID-19 infection.

[USE ACTIVE LISTENING, HEALTH COACHING AND INTERVIEW SKILLS TO RESPOND TO INFORMATION, ANSWERS AND COMMENTS THE CASE PROVIDES]

A. Symptoms and Hospitalization

Thank you for the information we've discussed so far. Now I need to ask you about symptoms, starting either before or after your test. This will help me determine the starting point for identifying others you spent time with during the period you may have been able to pass on the virus.

Have you had any symptoms of illness?

If NO: Just to be sure, I'm going to read through a list of symptoms and if you would, please answer yes if you have experienced the symptom or no if you have not. *[Symptoms are grouped together below. Read through them all, even if they say none. If yes to any from the list, proceed to ask the question below. If answer is no to all, skip to "Contact Overview" section – this differs from actual script but has been changed for this practice session]*

If YES: When was the first day you felt any symptoms at all? ***[Month, Day]***

[Can jog memory by calling out the day of the week (e.g. So that was Monday?)]

If unsure of start date: Please provide your best estimate. Even if these were minimal symptoms like being extra tired or having a runny nose, we want to be sure to capture the first day you felt symptoms.

Tell me about your symptoms.

[Allow case to name symptoms. Once case has finished naming symptoms on their own, read the list of remaining possible symptoms not mentioned then go back through list and verify.]

__ Cough, __ Difficulty breathing or shortness of breath?

__ Fever, __ Chills, __, or __ Muscle aches?

If yes to fever → What was your temperature? (__ >100.4F or __ Subjective)

If yes to chills → Did you also have shaking with the chills? (rigors)

__ Runny nose (rhinorrhea), __ Sore throat, __ Headache, __ Loss of taste or __ Smell?

__ Nausea, __ Vomiting, __ Abdominal pain, or __ Diarrhea?

__ Rash/Skin changes (dermatologic), __ Blood clot or Stroke (thromboses)

__ Other: ____________________; __ None

Are you still having symptoms? *If no, indicate the date symptoms resolved.*

Have you ever needed to stay overnight at the hospital during your illness?

If no, select "no" and proceed to next section.

If yes, select "yes" then fill in the hospital details (hospital name, dates of hospitalization and discharge, if patient was admitted to the ICU).

B. Contact Overview

One of the most important things that we can do as a community to stop the epidemic in *[county the person is located in]* is to identify people who may have been exposed to the virus. We need your help to do this.

We are talking to everyone who has been diagnosed with COVID-19, such as yourself. We need to notify individuals you came into close contact with while you were contagious and urge them to get tested. This will help them protect themselves and their families; we want to make sure they receive the help they need to do that.

During this process we do everything we can to keep your diagnosis confidential. Sometimes that may not be possible, for example, if you were involved with an activity where there was only one other person present. I also want to assure you that your information is kept confidential and protected by California's strict privacy law.

What questions do you have before we move on to the next part of our discussion?

C. Creating New Contacts

[This script provides an example of how to obtain contacts in a comprehensive way.]

So your symptoms started on ***[Month, Day]***. Let's think through everyone you have been around since 2 days before that, meaning ***[Month, Day]***.

If no symptoms, use 2 days before the date the first positive test was obtained.

I want you to think about people you are around routinely in your daily life: your family, friends, coworkers, plus any other people you may have been in close contact with, including intimate or sexual partners. We will also discuss anyone you've been in close contact with – which means within 6 feet (2 meters) of for 15 minutes or more.

Please let me know if any of the people we talk about don't speak English as their first language.

[All questions are in reference to the Contact Elicitation Window = 2 days before onset of symptoms until isolation started; if asymptomatic, 2 days before the day the first positive test was obtained until isolation started.]

- **Household members or close friends:** Let's start with family members and friends.
  - Who lives in your household?
  - Who from your family has visited you at home or other places?
  - Of those we've just talked about, do you know if any:

    Have __ Diabetes, __ Heart, __ Lung, __ Kidney, or __ Liver disease?

    Are taking __ immunosuppressive medications such as steroids, __ Chemotherapy, __ following organ transplant, or __ have any other immunosuppressive condition *(such as lupus or HIV)*?

    Do you know if any are pregnant?

    Are any 65 years of age or older?

    If yes → Do they live in a long term care or skilled nursing facility?

- **Workplace:** Have you been working at a job site? *If yes* → Let's go through your coworkers one by one.
  - Who do you share a workspace with?
  - Who do you have regular meetings with?
  - Who is your boss?
  - Who reports to you?
  - Who do you eat meals with when you're at work?
  - Let's now go through others you interacted with at work one by one:
    - Who are the customers or clients you know by name?
  - How do you travel to work?
  - Do you have another job you've been to? ***If yes,*** *repeat work questions*

- **Social Events:** Let's look at your calendar together and think through social or recreational things you may have been a part of since ***[Month, Day]***. I am going to run through a list of activities. Please stop me if you have taken part in any of the following:
  - Had a meal at anyone else's house or had someone to your house for a meal?
  - Met with any friends to go shopping, play sports with, or for other fun activities?
  - Went to a place of worship, a restaurant, any gatherings, parties, or social events?
  - Went to the gym or exercise class, sporting event, concert, or other event where there were a lot of people?
  - Traveled (car road trips, bus, trains, air travel)?
  - Had visitors from out of town?
- Have you been to a school? *If yes →* Tell me about that.
- Have you been to a health appointment or health facility (other than where you got your test for COVID- 19)?
- Have you had any intimate partners?

Collect detailed contact information on anyone who had close contact (more than 15 minutes) and close proximity (less than 6 feet) ***beginning two days prior to first feeling sick*** *(or if no symptoms, 2 days before the day the first positive test was obtained.* ***Make sure to include the date of last exposure*** *or their best estimate if they are unsure.*

For each of the close contacts that we've identified together, I need their full name, age or date of birth, a phone number and email if you have it, address of residence, preferred language, and the date that you last spent time with them. This information is provided to a contact tracer who will reach out to them, just like I did with you. Your name will not be provided to your close contacts unless you notify them yourself. Is it ok to proceed?

- First Name,
- Last Name
- Type of contact *(household, close friend/associate, workplace, healthcare setting, air/cruise travel, community, other, unknown, relative, not same household.)*
  - If household – Household relationship (spouse/partner, child, parent, grandparent, friend, colleague, roommate, sibling, other)
- Ever symptomatic? (yes, no, unknown)
- Age (Date of birth if known)
- Phone number
- Email Address
- Address – City, State/Province, zip code/postal code, country, and County
- Do you know if the person is immunocompromised or pregnant? (yes, no, unknown)
- Occupation
- Is the person experiencing any symptoms?
- Language preference
- Date of last exposure

D. Informing Contacts

I would like to encourage you to let people know about their exposure. It would also be very helpful, if you can let them know that the health department will be calling, so they might be more likely to answer their phone. If you are not comfortable with this, we understand. We will reach out to them regardless.

It's important we speak with them for a few reasons. **First**, if they are having symptoms they likely need to get testing for COVID-19 and we can help them get a free, confidential test. **Second**, we want to help them protect their friends and family by ensuring they know how to prevent spreading it to others in case they have it.

I know that all of this can sound scary, but we also know that the only way to beat this pandemic is to work together as a community to stop it. The more contacts we can find and help get tested early, the more cases of COVID-19 we can prevent in the future to keep California open.

If for some reason you didn't feel comfortable telling us all the people you were in close contacts with or if you remember some contacts after our call, you can also let them know yourself. You can even tell them anonymous by text or e-mail using the website tellyourcontacts.org.

[PROVIDE CHECK-IN QUESTIONS/SUMMARIZE AS APPROPRIATE]

STOP ROLE PLAY AND DEBRIEF

Contact Tracer Interview Practice –

DAY 2 Skills Lab Learner Guide

Activity 1

PURPOSE: To provide a contact tracer with an opportunity to practice conducting a Contact Tracing Interview.

INSTRUCTIONS

Part I: Each person will have **5 minutes** to practice the **Introduction** and **Demographic and Contact Information** sections of the Contact Tracing Interview Script, which will include addressing one of the contact's concerns listed below. One person will play the role of the COVID-19 Contact Tracer and one person will play the role of the COVID-19 Contact.

COVID-19 Contact Tracer: Today's date is May 6 and you have just received a line list of contacts to phone. You will be calling John Morales to inform him of his exposure to COVID-19. His date of birth is Jan. 22, 1997. Use the script as your guide and be prepared to address any concerns he may have.

COVID-19 Contact: You are John Morales. Your date of birth is Jan. 22, 1997. You identify as male. You will have one of the concerns listed below. You know you have been exposed to a case named Philip Brown, your housemate. The last date of exposure to Philip is May 5. You speak English, you have no symptoms or underlying medical conditions. **Make up all other information when you play John.**

Each person will pick a Contact Tracer number to determine the order in which you practice playing the role of the tracer. Use the chart below to have the Contact express a concern as listed below when you practice. Rotate who will play the role of the Contact.

CONTACT TRACER	CONTACT CONCERN
#1	Why do you need my date of birth?
#2	Is this a scam?
#3	Do I have to talk to you?
#4	Why do you need to know my race?
#5	Why are you asking me about my sexual orientation and gender identity?

DEBRIEF: After everyone has practiced, the facilitator will provide their observations and constructive feedback.

Activity 2

In your same groups you will now practice a **different section** of the script. One person will play the role of the Contact Tracer and one person will play the role of the COVID-19 Contact.

The Contact Tracer will read the bridge phrase for their selected section and be prepared to address one of the concerns below

As a Contact you will have a unique concern related to a section of the Contact Interview Script as described below.

Use your same Contact Tracer number to determine the order of the role play. Use the chart below to have the Contact express a concern as listed below when you practice. Rotate who will play the role of the Contact.

CONTACT TRACER	SECTION	CONTACT CONCERN
#1	Symptoms	"I feel fine, I don't have any symptoms"
#2	Medical Conditions	"I don't have medical insurance, where can I go to get tested?"
#3	Exposure/ Living Situations	"I live in a small studio apartment with my roommate"
#4	Quarantine/ Isolation Assessment	"What if I never develop symptoms? Will I have to quarantine for the entire 14 days?"
#5	Key Messages/ Closing	"If I test positive for coronavirus, can I get it again?"

DEBRIEF: After everyone has practiced, the facilitator will provide their observations and constructive feedback.

Contact Tracer Interview Practice –

DAY 3 Skills Lab Learner Guide

Activity 1

PURPOSE: To provide the contact tracers with an opportunity to consider what tools they will use to build rapport throughout the interviewing process.

PART 1: EXPRESSING EMPATHY & APPRECIATION

Below you will read a response from the contact to a question the contract tracer has asked. Your task is to come up with your next line using one of the following active listening techniques: **affirmation, reflective statement, or open-ended question.**

1. **After asking, "What do you know about COVID-19?"**
   The person sounds sad. He shares with you that his grandfather is currently in the hospital due to COVID-19 and tells you that it is hard for him to think about his own health right now.
   Your next line: ______________________________

2. **After asking, "What concerns do you have about COVID-19?"**
   The person responds: "It's all over the news. I'm definitely knowledgeable and wear a mask whenever I go out."
   Your next line: ______________________________

3. **When asking about the contact's living situation,**
   The client states they live with a partner in a studio apartment and is more concerned about their ability to pay rent than being exposed to COVID-19. A lot has changed since this all began.
   Your next line: ______________________________

4. **After giving someone a recommendation to quarantine,**
   The person says they are worried about their parents. They feel close to them and don't feel that they can stay away from them for the full quarantine period. At the same time, they do not want to do anything to put them at risk.
   Your next line: ______________________________

Contact Tracer Interview Practice –

DAY 3 Skills Lab Learner Guide

Activity 2

PURPOSE: To practice health coaching skills in the context of speaking to contacts about COVID-19. ***We are focusing exclusively on practicing the skill in this section. You do NOT need to use your script at this time or to role-play an entire phone call.***

INSTRUCTIONS FOR EACH ROLE PLAY

1. For each scenario, select one person to play the contact tracer and one person to play the contact. The contact tracer may use the checklist below to help them during the role-play.
2. Take no more than **5 minutes** to role-play each scenario.
3. After the role-play, take no more than **3 minutes** to provide feedback. What did the contact tracer do well? What might the contact tracer do better? Use the checklist after the scenario to guide your discussion.
4. Choose new people to play the roles of contact tracer and contact. Repeat the scenario.

PRACTICE PART 1: SETTING THE AGENDA

Contact Tracer/Case Investigator: You are speaking to a new contact and have already introduced yourself. Now you want to set the agenda for the call. For this scenario, your only task is to set the agenda/plan for the call.

Let the contact know that during your call you would like to talk about the following items:

- Symptoms, including any symptoms the contact is currently experiencing or may experience
- The client's needs for support with food, prescriptions, or other things

Contact: When the contact tracer asks what you want to talk about, you share the following:

- I need to pick up my prescription – what do I do?

SETTING THE AGENDA CHECKLIST	
☐ Asks contact what they want to be sure that you discuss	*I have a few things that I hope to talk about today. I also want to be sure we talk about anything on your mind. What concerns or questions do you want to talk about today?*
☐ Shares things that contact tracer/case investigator wants to talk about	*I would also like to talk about…*
☐ Identify what from the contact's concerns you can help address today and what you need help in answering.	*We will be sure to talk about your prescriptions today.*
☐ Briefly summarize all of the agenda items: yours and the contact's	*Okay, so how about if we start with how to get your prescriptions and then cover symptoms and other resources you may need?*

Observations:

PRACTICE PART 2: ASK-TELL-ASK AND CLOSING THE LOOP - QUARANTINE

Contact Tracer/Case Investigator: Use Ask-Tell-Ask and Closing the Loop to assess and build on your contact's knowledge of how to quarantine.

Make sure your contact knows that quarantine precautions include:

- Staying at home except for urgent medical visits
- Avoiding contact with other people, especially those in high risk groups
- Avoiding sharing personal household items with others in your house
- Using separate bathroom or wiping down surfaces with disinfectant after use

REMEMBER: YOU DO NOT NEED TO ROLE PLAY AN ENTIRE PHONE CALL OR TO MAKE AN ACTION PLAN!

Contact: You know some, but not all, quarantine recommendations.

ASK-TELL-ASK & CLOSING THE LOOP: OBSERVATION CHECKLIST	
☐ Asks permission to talk about how to prevent the spread of coronavirus	*Would it be okay to talk about what quarantine means?*
☐ Asks open-ended questions to assess knowledge	*What have you heard about what you need to do when you quarantine?*
☐ Provides information or advice ONLY when the contact asks or doesn't know	*That's great! [Confirm information that is correct.] A few other things that public health experts recommend are…*
☐ Asks contact to restate new information in their own words (closes the loop)	*We've covered a lot of information today. To make sure I didn't miss anything important, would you mind describing in your own words what quarantine means?*
Observations and suggestions for improvement?	

PRACTICE PART 3: ACTION PLANNING

Contact Tracer/Case Investigator: You have already introduced yourself to the contact and talked about the basics of COVID-19, prevention, and symptoms. Your contact has a specific concern that makes quarantine challenging. Use action-planning skills to help the contact/client identify the first steps that they can take to address their concern.

START WITH THE QUESTION: What do you think may make it difficult for you to quarantine?

Contact: Select one of the following prompts:

- You usually check on an aging parent a few times a week and bring them groceries.
- You need a refill on your asthma inhaler.
- You really miss your friends and don't know how you are going to handle this.

ACTION PLANNING: OBSERVATION CHECKLIST	
☐ Ask permission to talk about the challenge together	*It sounds like you are concerned about X. Would it be okay to talk more about ways to…*
☐ Get a menu of options on the table<br>○ Ask about what the contact is already considering<br>○ Share what other people do	*Some of the things that I hear other people do to get their prescriptions include…*<br>*What do you think might work best for you?*
☐ Ask open-ended questions to get specific.<br>○ What (First step)<br>○ How<br>○ When<br>○ Who	*What is the most important thing to do first?*<br>*Who might be able to help you with that?*<br>*When will you do that?*
☐ Asks the contact about their confidence	*On a scale of 0-10, where 0 is not at all confident and 10 is very confident, how confident are you that you will be able to carry out this plan?*<br>*[If below 7]: How might you change your plan to get to a 7?*
☐ Ask if it's okay to follow up	*Would it be okay for me to call you in a few days to see how that's going?*
☐ Close the loop	*We've talked about a lot of information and plans today! Would you mind describing in your own words what your plan is?*
Observations and suggestions for improvement?	
